# Grant Report on the Effects of Childhood Maltreatment on Neurocircuitry in Adolescent Depression

**DOI:** 10.20900/jpbs.20200016

**Published:** 2020-07-08

**Authors:** Marie L. Gillespie, Uma Rao

**Affiliations:** 1 Department of Psychiatry & Human Behavior, School of Medicine, University of California, Irvine, 5251 California Ave., Suite 240, Irvine, CA 92617, USA; 2 Department of Pediatrics, School of Medicine, University of California, Irvine, 333 The City Blvd. West, Suite 800, Orange, CA 92868, USA; 3 Center for the Neurobiology of Learning and Memory, University of California, Irvine, 309 Qureshey Research Lab, Irvine, CA 92697, USA; 4 Children’s Hospital of Orange County, 1201 W La Veta Ave, Orange, CA 92868, USA

**Keywords:** adolescence, maltreatment, depression, neurocircuitry, fronto-limbic, front-striatal, functional connectivity

## Abstract

This report describes the protocol for an ongoing project funded by the National Institutes of Health (R01MH108155) that is focused on effects of childhood maltreatment (MALTX) on neurocircuitry changes associated with adolescent major depressive disorder (MDD). Extant clinical and neuroimaging literature on MDD is reviewed, which has relied on heterogeneous samples that do not parse out the unique contribution of MALTX on neurobiological changes in MDD. Employing a 2 × 2 study design (controls with no MALTX or MDD, MALTX only, MDD only, and MDD + MALTX), and based on a cohesive theoretical model that incorporates behavioral, cognitive and neurobiological domains, we describe the multi-modal neuroimaging techniques used to test whether structural and functional alterations in the fronto-limbic and fronto-striatal circuits associated with adolescent MDD are moderated by MALTX. We hypothesize that MDD + MALTX youth will show alterations in the fronto-limbic circuit, with reduced connectivity between the amygdala (AMG) and the prefrontal cortex (PFC), as the AMG is sensitive to stress/threat during development. Participants with MDD will exhibit increased functional connectivity between the AMG and PFC due to self-referential negative emotions. Lastly, MDD + MALTX will only show changes in motivational/anticipatory aspects of the fronto-striatal circuit, and MDD will exhibit changes in motivational and consummatory/outcome aspects of reward-processing. Our goal is to identify distinct neural substrates associated with MDD due to MALTX compared to other causes, as these markers could be used to more effectively predict treatment outcome, index treatment response, and facilitate alternative treatments for adolescents who do not respond well to traditional approaches.

## INTRODUCTION

Major depressive disorder (MDD) is the leading cause of disability worldwide [1,2], and it frequently emerges during adolescence [3]. Adverse experiences that occur in early childhood, such as various types of abuse/maltreatment (MALTX), are common risk factors for the development of MDD, accounting for up to 50% of attributable risk [4–6]. However, it is important to distinguish between depressed individuals with and without MALTX history, as critical clinical differences (e.g., age of onset, symptom presentation, treatment response, clinical course) suggest the presence of two distinct subtypes [7–11]. Findings from adult neuroimaging studies may be influenced by the presence of MALTX within heterogeneous samples, with abuse history likely accounting for some structural and functional differences between adults with MDD and healthy controls (e.g., reduced hippocampal [HIPP] size and amygdala [AMG] hyper-reactivity) [11–13]. It is unclear whether MALTX is a qualitatively different type of stressor compared to other psychosocial stressors that play an etiological role in the development of MDD. Our ongoing study addresses this gap within the literature by applying a cohesive theoretical model incorporating behavioral, cognitive, and neural domains.

The overarching aim of this study is to determine whether two distinct MDD subtypes (with and without MALTX) are warranted through an examination of structural and functional neural circuits in a diverse sample of adolescents. Because adolescence is characterized as the period with the most pronounced neural changes and maturation in grey and white matter since infancy [14–17], and that the brain-based effects of MALTX appear at this stage [18–20], we focus on 13- to 17-year-old participants. Primary hypotheses focus on the neural circuits implicated in cognitive-emotional (fronto-limbic) and motivational-reward (fronto-striatal) processes, as they play a central role in the core depressive symptoms (i.e., negative mood and anhedonia; Diagnostic and Statistical Manual of Mental Disorders, 5th ed.; DSM–5) [21]. Using both dimensional and categorical perspectives, as well as multi-modal imaging techniques, neural alterations associated with depressive symptom profiles will be examined. Although inclusion and exclusion criteria for our study will be described in further detail in the methods section, for the purposes of describing study aims, hypotheses, and background, the following group designations will be used heretofore: MDD (i.e., adolescents meeting DSM-5 criteria for current MDD; no history of abuse prior to age 10); MALTX (i.e., adolescents with history of physical, sexual, and/or emotional abuse prior to age 10; no psychiatric history); MDD + MALTX (i.e., adolescents with current MDD and a history of abuse prior to age 10); and NC (i.e., normal controls/adolescents with no personal or family history of a psychiatric disorder, and no abuse history).

### Aims, Hypotheses, and Background

Aim 1: Test whether structural and functional alterations associated with depression in the fronto-limbic circuit are moderated by a history of abuse and identify structural and functional differences in this circuit between MDD and MDD + MALTX. Structural differences: We hypothesize that MDD + MALTX will have smaller grey matter volume in the HIPP and prefrontal cortex (PFC) but greater AMG volume, and lower fractional anisotropy (FA) in the fronto-limbic tracts (cingulum/uncinate fasciculus), compared to MDD. Functional differences: MDD + MALTX will exhibit reduced functional connectivity between the AMG, HIPP, and PFC, both at rest and in response to a cognitive-emotional task, and MDD will exhibit increased functional connectivity between these regions.

Aim 2: Test whether structural and functional alterations associated with depression in the fronto-striatal circuit are moderated by a history of abuse and identify structural and functional differences in this circuit between MDD and MDD + MALTX. Structural differences: We hypothesize that MDD will have smaller grey matter volume in ventral striatum (VS) and lower FA in the fronto-striatal tracts compared to MDD + MALTX. Functional differences: MDD will exhibit increased functional connectivity between the VS and PFC during resting-state as well as reward anticipation and receipt phases in response to a reward-processing task. Additionally, MDD + MALTX will exhibit reduced functional connectivity at rest and only during the anticipation phase of the reward-processing task.

Aim 3: Test whether abuse history moderates the associations of specific symptom constructs, such as negative or positive affect, anhedonia, or rumination, with key nodes in the fronto-limbic and fronto-striatal circuits. We hypothesize that a history of abuse will moderate the associations of negative affect and rumination with fronto-limbic circuit, and anhedonia with fronto-striatal circuit. However, we expect that abuse history will not moderate the association between positive affect and fronto-striatal circuit.

**Figure 1.**
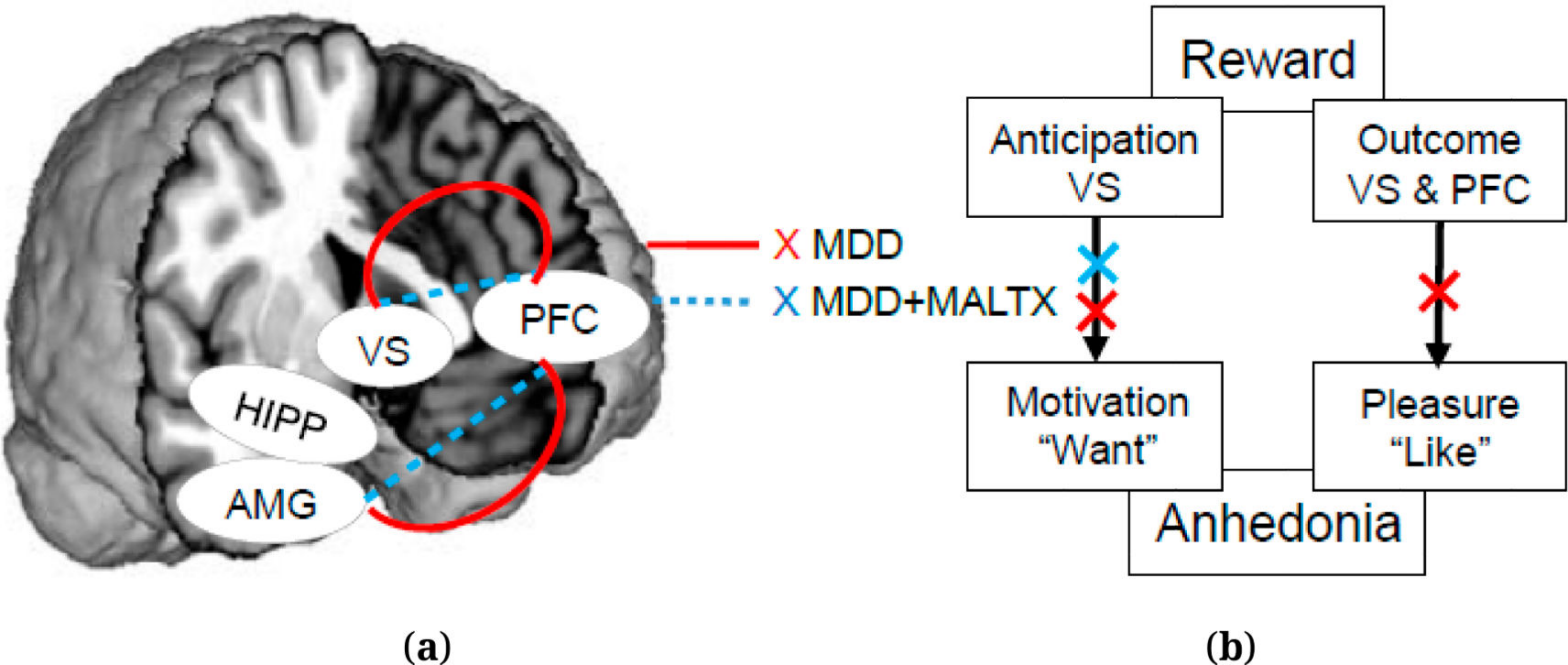
Neural Deficits in MDD and MDD + MALTX (**a**) Left (brain): Fronto-limbic and Fronto-striatal Circuits; (**b**) Right (explanatory model): Anhedonia and Reward-Processing.

Figure 1 (a and b) presents the explanatory model described in the hypotheses with respect to fronto-limbic and fronto-striatal circuits in MDD and MDD + MALTX. With guidance from seminal adult and pediatric neuroimaging studies [20,22–35], our model hones in on key structures detailed in the literature focusing on samples with either abuse or MDD, as very few studies stratified MDD with and without MALTX in their samples [36]. We conceptualize and parse out history of abuse as a unique stressor compounding the development of depression, and therefore, highlight the crucial role of the fronto-limbic circuit, which comprises brain regions that are highly reactive to threat and susceptible to the impact of stress. For example, the AMG plays a central role in emotion-processing and is implicated in the rapid processing of threat-related stimuli. The PFC modulates AMG response through inhibitory regulation in non-threatening situations. Honing in on PFC sub-regions that may be implicated in these processes, we identify the ventromedial PFC (vmPFC) and dorsolateral PFC (dlPFC) as areas of interest, as these regions are salient to stress response and cognitive-emotional regulation processes [37,38]. The HIPP similarly processes threat- and non-threat-based stimuli and facilitates the retrieval of explicit memories. Early childhood abuse experiences essentially interrupt and reprogram the development of salient components of the fronto-limbic system, such that the AMG interprets non-threatening events as threatening, thereby yielding globally exaggerated responses [33,39]. Further, the PFC, specifically the vmPFC and dlPFC components, is ineffective in down-regulating this AMG response with reduced functional connectivity between these brain regions (blue line, Figure 1a), leading to emotional dysregulation and depression [20,22,23,33,40,41]. However, in depressed samples without the interplay of abuse history, self-referential, ruminative and negative thoughts may predominate instead of threat-based reactions, with PFC hyper-response and increased functional connectivity between the AMG and PFC (red line, Figure 1a) [37,42,43].

Figure 1b exemplifies two distinct behavioral components linked to anhedonia based on translational study findings—the motivational (“wanting” reward) and consummatory (“liking” reward/experiencing pleasure)—linked to individual reward processes and associated neural substrates (ventral striatum [VS] implicated in the anticipatory/motivational component; and both VS and PFC implicated in the outcome/consummatory component) [44]. The chronic stress experienced by individuals with exposure to childhood abuse is understood to increase risk for the development of apathy/depression, thereby affecting the motivational component of the reward/fronto-striatal circuit (blue-cross, Figure 1b). In non-abused MDD samples, decreased capacity to experience pleasure is a prominent feature in addition to reduced motivation, and both components of the reward/fronto-striatal circuit may be affected (red-cross, Figure 1b). Lastly, reward-processing and decision-making also trigger socioemotional processes; fronto-limbic and fronto-striatal circuits may therefore be *interconnected*. This dynamic, which simultaneously influences emotional and reward processes, leads to decreased functional connectivity in MDD + MALTX and increased connectivity in MDD in both circuits (Figure 1a) [19,20,22–18,31,33].

### Significance of the Research and Clinical Implications

The public health impact and economic burden associated with adolescent MDD are well-recognized [1–3,45]. Adverse childhood experiences and early-life stress significantly increase risk for the development of depressive disorders; more than one-half of MDD cases are estimated to be linked to MALTX [46], with individuals exposed to early sexual, physical, and/or emotional abuse being 2.5 times more likely to be diagnosed with MDD in childhood or adolescence [47]. The strong association between MALTX and MDD has also been found to emerge in various stages of adulthood [7,41,48]. Indeed, while maltreatment occurs most commonly in early childhood [49], the impact it has on psychological and brain functioning often appears years later during the salient developmental period of adolescence [50,51]. For instance, several studies have found reduced HIPP size in adults, but not children, who experienced early abuse [13,19,35], with researchers indicating that maltreatment-related HIPP alterations frequently emerge in adolescence [17,52,53].

Incidence rates of MDD in adolescence have been estimated from 11% to 25% [3,47]. However, researchers have yet to make a clear distinction between MDD with and without MALTX, with studies reporting on differences based on post-hoc analyses in heterogeneous samples [11–13,54], despite data indicating that these are separate subtypes of depression with respect to clinical course, symptom presentation, treatment responsiveness, and prognostic outcome [7,8,10,55].

Moreover, genetic and neurobiological studies have posited that depressed individuals with and without MALTX have different neurobiological substrates [11–13,30,34,36,56–58], however, the nature of these differences has not been well characterized. Of note, one of the most well-established markers of MDD in adult neuroimaging studies has been reduced HIPP volume and AMG hyper-reactivity, yet these findings were present only in the subset of MDD patients who also had MALTX, while MDD patients without MALTX yielded similar profiles to those of normal/healthy controls [11,12]. Additionally, pure MALTX samples (i.e., no psychiatric disorder) also exhibited reduced HIPP volume and AMG hyper-reactivity, suggesting that these alterations are driven by MALTX and present additive neurobiological risk factors for MDD [13,19,22,23,33]. Of note, a small-scale adolescent-based study found that reduced HIPP volume was associated with MALTX in both MDD patients and healthy controls, but that amygdala reactivity was not associated with MALTX [59].

The treatment and prognostic implications of identifying and differentiating the biological bases and neural correlates of these two depression subtypes include the opportunity to shape clinical formulation and strengthen intervention guidelines. Our ongoing study is making efforts to identify distinct neural substrates associated with MDD in MALTX victims, as these specific markers can be used to effectively predict treatment outcome [60,61] and more accurately index treatment response [62–64]. In doing so, we hope to facilitate the use of alternative treatments for individuals who do not respond well to traditional approaches. For example, findings from some clinical trials have suggested that depressed individuals respond differentially to traditional antidepressant treatments based on their childhood abuse histories [8,10,65,66]. In MDD + MALTX samples, who are prone to threat-based cognitive-emotional processing, pharmacological agents that attenuate AMG hyper-reactivity in response to threat-based stimuli (e.g., anxiolytics, cannabinoid receptor agonists) [67,68] may be more effective than selective serotonin reuptake inhibitors (SSRIs), as these agents tend to increase short-term anxiety through enhanced effects on the acquisition and expression of fear conditioning [69,70]. Additionally, neurofeedback interventions may be used to individually regulate the AMG in the two MDD subtypes, for instance, by down-regulating during the presentation of threat-based stimuli in MDD + MALTX rather than up-regulating during recall of positive autobiographical memories in pure MDD samples [71–74]. Correspondingly, if motivational anhedonia/reward-processing predominates the pathophysiology of MDD + MALTX, it may be more effective to integrate behavioral activation interventions in conjunction with more traditional cognitive-behavioral therapy [8,10,56,75–77].

We identify several shortcomings in the literature and present novel approaches to address these gaps, with the aim of distinguishing between the additive and interactive effects of MALTX and identifying distinct MDD subtypes that would inform good clinical practice. First, we address the methodological limitations of neuroimaging studies that focused only on adult, heterogeneous samples of depression by stratifying adolescent samples based on rigorous criteria of MDD and MALTX. By focusing on an adolescent sample, we can also minimize the confounding effects that recurrent episodes and treatments can have on the brain, as many adolescents are more likely to be in their first depressive episode and treatment-naïve. Accordingly, we also screen out for the potentially confounding effect of psychotropic medications. Second, we comprehensively assess depressive symptoms and maltreatment history through multi-modal, multi-informant assessments. This study implements validated, clinician-led psychodiagnostic batteries and structured interviews with both adolescents and caregivers to determine group stratification, as many studies have been limited to retrospective self-reports of adverse childhood experiences and depressive symptoms.

Third, studies focusing on neuroimaging changes associated with MALTX have primarily relied on unimodal procedures [13,19] and we address this limitation by implementing multi-modal imaging techniques, including macro- and micro-structural, as well as resting-state and task-based functional MRI scans. In post-hoc analyses, we will explore how structural alterations relate to functional changes [78–80]. These methods will enhance our knowledge-base on pathophysiology and potentially contribute to improved treatment guidelines and prognosis forecasting [55–59].

While studies focusing on single brain regions have been essential in helping to identify regions of interest for this population [19,31], we test the aforementioned explanatory model (see Figure 1) by taking a multiple circuit approach which hones in on mechanisms specifically implicated in core depressive symptomology (i.e., fronto-limbic circuit for negative mood and fronto-striatal circuit for anhedonia). Given that neural events rarely occur in isolation, and as maltreatment and depression impact multiple brain regions, a systems-level analysis increases our understanding of the existing neural models [13,19,27,31]. Further, there are significant benefits to using combined structural and functional neuroimaging methodology, particularly when aiming to understand the mechanisms at play in the pathophysiology of depression. For instance, one meta-analysis examined brain changes associated with adult depression and found under-activation of different parts of the dlPFC depending on scanning methodology (i.e., resting-state vs. task-based) [31]. Lastly, our approach capitalizes on the Research Domain Criteria (RDoC) [81,82] by examining the neural substrates of positive and negative valence systems alongside broadly defined clinical categories and efficiently builds upon our current knowledge-base of the neurobiology of depression.

## METHODS

Our study employs a 2 × 2 design to test a depression x maltreatment interaction hypothesis and ensuring that main effects of MALTX are not misinterpreted as simple main effects of MALTX for the MDD groups. We aim to enroll 240 participants equally distributed across four groups (MDD + MALTX; MDD; MALTX, and NC), which are group-matched on age, sex, pubertal stage, race/ethnicity and socioeconomic status (SES).

### Recruitment and Eligibility

All human subjects research described herein was approved by the Institutional Review Board at University of California, Irvine in 2017 (Protocol #2017–3440). Following a NIH (Sponsor) initial site-visit and approval, recruitment was initiated in February 2018. Participants are recruited from communities throughout Southern California. Adolescents of both sexes/genders and all racial/ethnic groups are eligible to participate if they are between 13 and 17 years of age and in Tanner Stage II or greater of pubertal development. Exclusion criteria include contraindications for imaging procedures (e.g., metallic devices, claustrophobia), as well as conditions that would affect brain development, including IQ below 80, birth complications or premature birth, maternal substance abuse during pregnancy, neurological disease, or head trauma with loss of consciousness. As previously mentioned, youth taking psychotropic medication that may affect the central nervous system are excluded, unless willing to adhere to standard wash-out periods for certain medications (e.g., stimulants for attention-deficit hyperactivity disorder) prior to scanning. Youth who used alcohol and/or drugs in the week prior to study entry (based on self-report or urine drug screen), are suspected to be pregnant, and those who reported experiencing multiple unrelated forms of trauma (e.g., natural disaster, accidents, gang violence) are not eligible.

Furthermore, participants and/or their biological parents with a history of mania or hypomania are excluded, as unipolar and bipolar depression may have distinct neural markers. To further minimize diagnostic comorbidity, youth are excluded if they meet DSM-5 criteria for moderateto-severe disruptive disorders, substance use disorders or autism spectrum disorder in the previous six months, or were exhibiting psychotic symptoms or active suicidal ideation at recruitment. However, youth determined to meet criteria for anxiety and trauma-based disorders (e.g., post-traumatic stress disorder; PTSD) are eligible, as these diagnoses are often comorbid with MDD, have overlapping symptoms, and likely have shared etiological factors [83–86]. We will identify the neural correlates of MDD in youth with and without PTSD or anxiety disorders in exploratory analyses [87].

### Assessments and Group Stratification

The assessment schedule is presented in Table 1. Following the administration of phone screens to determine eligibility criteria, adolescents and a parent/legal guardian attend the initial lab visit (Visit 1) during which informed consent from the parent and assent from the youth are obtained. Participants subsequently complete demographic questionnaires and pubertal status ratings (i.e., Tanner Stages), as well as comprehensive psychodiagnostic batteries and structured interviews to determine group classification.

Youth and caregivers are separately administered the computerized version of the Kiddie Schedule for Affective Disorders and Schizophrenia—Present and Lifetime Version (K-SADS-COMP V2.0; K-SADS-PL) [88] by post-doctoral clinical psychologists, supervised by the Principal Investigator (PI) who is a licensed child and adolescent psychiatrist. Spanish-speaking parents are administered the 1.0 Spanish computerized version by a Spanish-speaking clinician. The K-SADS-PL/K-SADS-COMP is a DSM-5-based semi-structured interview that assesses for symptom onset, course, duration, severity, and impairment in order to ascertain present and lifetime history of psychiatric disorders. Diagnosis is subsequently determined by using a consensus of child and caregiver interviews. The web-based version has strong convergent validity and high inter-rater reliability for depressive disorders [88].

The Childhood Adversity Interview (CAI) [89] is also a semi-structured interview administered individually to adolescents and caregivers. This instrument focuses on various types of adversities (i.e., separation and loss of primary caretaker(s), life-threatening illness/injury to self or others, witnessing domestic violence) and maltreatment experiences (i.e., emotional abuse, physical abuse, sexual abuse, and neglect) [90]. Taking into account contextual factors and circumstances, interviewers use information from both informants to determine severity of adversity in each domain on a 5-point scale (1 = no adversity; 3 = moderate; 5 = extreme). The CAI was modified from the Childhood Trauma Interview [91], and has shown good inter-rater and test-retest reliability [92]. Previous studies have shown good discriminative power to detect neurobiological differences in adolescents by using a threshold score of ≥3 on the three abuse items (emotional, physical and sexual abuse) compared to scores of 1 on abuse, neglect and domestic violence items for those without MALTX [53,90,93]. As the current study aims to understand the enduring, long-term effects of maltreatment history on neurobiology, adolescents are eligible for the MALTX groups if they meet threshold criteria for abuse occurring prior to age 10. Scores are calculated for both lifetime abuse as well as abuse experienced prior to age 10. Exploratory analyses will compare MALTX groups with abuse only prior to age 10 and those that also had abuse after age 10 years. Further, although we will be examining all adverse experiences in exploratory analyses, our primary focus is on the aforementioned abuse items, as these incidents are more reliably documented.

Participants in the MDD + MALTX group meet DSM-5 criteria for current unipolar MDD based on K-SADS-COMP consensus ratings and meet the threshold for significant abuse history prior to age 10 based on ratings of ≥3 (at least moderate severity) on any of the three abuse items on the CAI. The MDD group meets criteria for current unipolar MDD and does not have any significant abuse history (score 1/none on each abuse, domestic violence, and neglect items on the CAI). Adolescents categorized in the MALTX group meet threshold criteria for significant history of abuse but do not have significant current or lifetime psychiatric history, with the exception of phobias. The NC group does not have any significant abuse or psychiatric history, as indicated above.

Parental psychopathology is assessed using a semi-structured interview, the Family History-Research Diagnostic Criteria (FH-RDC) [94], administered by post-doctoral clinical psychologists under the PI’s supervision, with the primary caregiver as the informant. For the MDD and MDD + MALTX groups, a current or past history of mania or hypomania in either of the biological parents is an exclusion. For NC, any major Axis I disorder on the FH-RDC is an exclusion.

Visit 1 assessments, as indicated in Table 1, also include clinician administration of the Children’s Depression Rating Scale–Revised Version (CDRS-R) to assess for symptom severity [95,96], and a self-reported depression scale (Beck Depression Inventory) [97]. Additional self-report measures include the Temporal Experience of Pleasure Scale (TEPS) to assess individual traits in anticipatory and consummatory experiences of pleasure [98], and the rumination subscale of the Children’s Response Style Questionnaire (CRSQ), which asks about participants’ use of self-focused thoughts regarding the causes and consequences of depressed mood [99]. Supplemental to clinician-determinations of abuse severity on the CAI, participants also complete MALTX experiences using the Childhood Trauma Questionnaire (CTQ) Short Form [100].

With respect to measures used in exploratory analyses, affect and mood (the Positive and Negative Affect Schedule) [101], parent and youth ratings on anxiety symptoms (Screen for Child Anxiety-Related Emotional Disorders) [102], PTSD symptomology (PTSD Checklist for DSM-5) [103], stress (Adolescent Stress Questionnaire) [104], social support (UCLA Social Support Inventory) [105] social functioning (Social Adjustment Scale-Self Report) [106] and parent-rated autism traits (Social and Communication Disorders Checklist) [107] are also assessed. Lastly, parent-child relationships are measured with the Parental Bonding Instrument [108], the Child-Parent Relationship Scale [109], and the parent- and youth-rated Questionnaire of Unpredictability in Childhood [110].

At Visit 1, a urine drug screen and MRI safety screen (to determine the presence of metallic devices and implants) are administered. Medication and treatment history are also gathered.

At visit 2, adolescents complete a neurocognitive battery to test whether executive functioning measures correlate with neural markers or psychiatric symptoms. The battery assesses for verbal and non-verbal reasoning (Vocabulary and Matrix Reasoning subtests of Wechsler abbreviated Scales of Intelligence) [111], working memory (N-Back Task) [112], inhibitory control (Color-Word Interference Subtest of Delis Kaplan Executive Function) [113], attention (Visual and Auditory Continuous Performance Test) [114], and parent- and youth-reported executive functioning (Behavior Rating Inventory of Executive Function) [115]. Participants also complete a mock scan to acquaint themselves with upcoming scanner environment and procedures, practice experimental fMRI tasks, and for researchers to assess for potential claustrophobia. Visit 3, often completed immediately following visit 2, is comprised of the neuroimaging tasks described in subsequent sections. Participants complete the same MRI safety screen that was completed at the initial visit to confirm the absence of metallic objects/devices and/or claustrophobic symptoms.

### Neuroimaging

The multi-modal imaging techniques include structural MRI (sMRI), diffusion tensor imaging (DTI), resting-state functional MRI (rs-fMRI) and task-based fMRI, all of which amount to approximately 90 min of scanning. Scans are acquired on a 3.0 Tesla Siemens Prisma (Erlangen, Germany) scanner, using a standard radiofrequency 12-channel head coil. T2-weighted scans, co-planar to the functional images (TR/TE = 6400/67 ms, FOV = 24 cm, matrix 256 × 256, flip angle = 149°) and high resolution T1-weighted scans (TR/TE = 2300/2.96 ms, FOV = 256 mm, 1 mm isotropic resolution, flip angle = 9°), are acquired for registration purposes. Functional images are acquired with a gradient-echo, EPI sequence: 34 oblique axial slices (4 mm thick, 1 mm gap), oriented to the AC-PC line, and encompassing the entire cerebrum and most of the cerebellum (TR/TE = 2000/25 ms, FOV = 24 cm, matrix = 64 × 64, flip angle = 77°). For rs-fMRI, which measures intrinsic functional connectivity, participants are instructed to remain awake, relax with their eyes open, and look at a fixation cross for approximately 10 min. For task-based fMRI scans, stimulus presentation is completed using E-Prime with the images projected onto an overhead LCD panel and a 5-button box for recording behavioral data. An automated higher-order shim procedure is applied to minimize magnetic field inhomogeneities.

### Structural MRI

**Figure 2.**
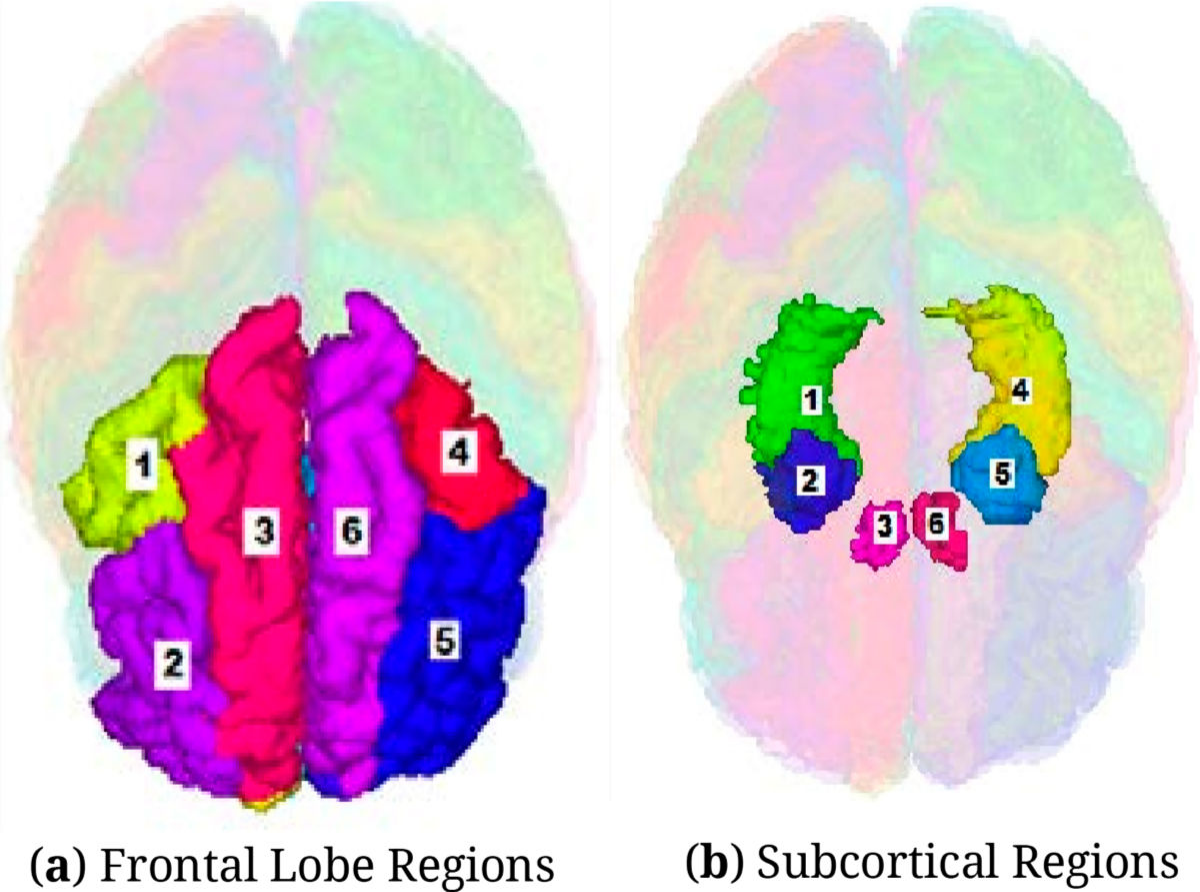
(**a**) Frontal Lobe Regions: Caudal Middle Left (1) and Right (4); Rostral Middle Left (2) and Right (5); Superior Left (3) and Right (6). (**b**) Subcortical Regions: Hippocampus Left (1) and Right (4); Amygdala Left (2) and Right (5); N. Accumbens Left (3) and Right (6).

**Figure 3.**
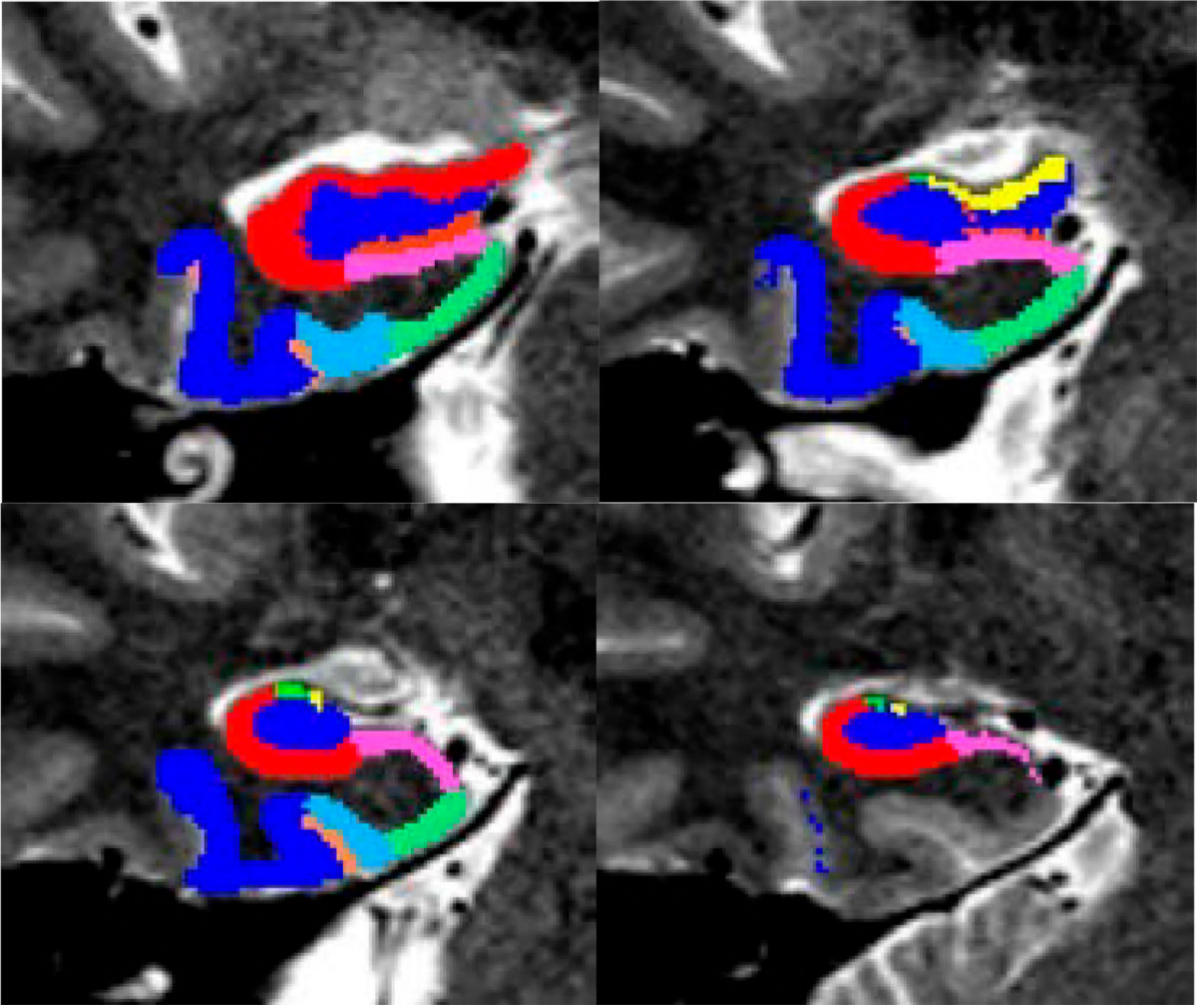
Right Hippocampus Sub-fields.

FreeSurfer image analysis suite is used to perform cortical reconstruction and volumetric segmentation [116]. Surface thickness and volumetric measures are extracted for each region of interest described in primary hypotheses (Figure 2a,b). The resulting maps are capable of detecting small differences between the groups. An experienced neuroimaging data-analyst performs quality assurance and manual review of all results on an ongoing basis, with monthly reports submitted to a primary supervisor and senior analyst. In secondary analysis, voxel-wise grey matter density (VBM) is compared using FSL-VBM [117], an optimized VBM protocol [118] carried out with FSL tools [119]. Using Automated Segmentation of Hippocampal Sub-field (ASHS) software [120–122], HIPP sub-fields are identified by fusing standard isotropic T1-weighted structural with high-resolution coronal T2-weighted data (Figure 3).

### Diffusion Tensor Imaging (DTI)

**Figure 4.**
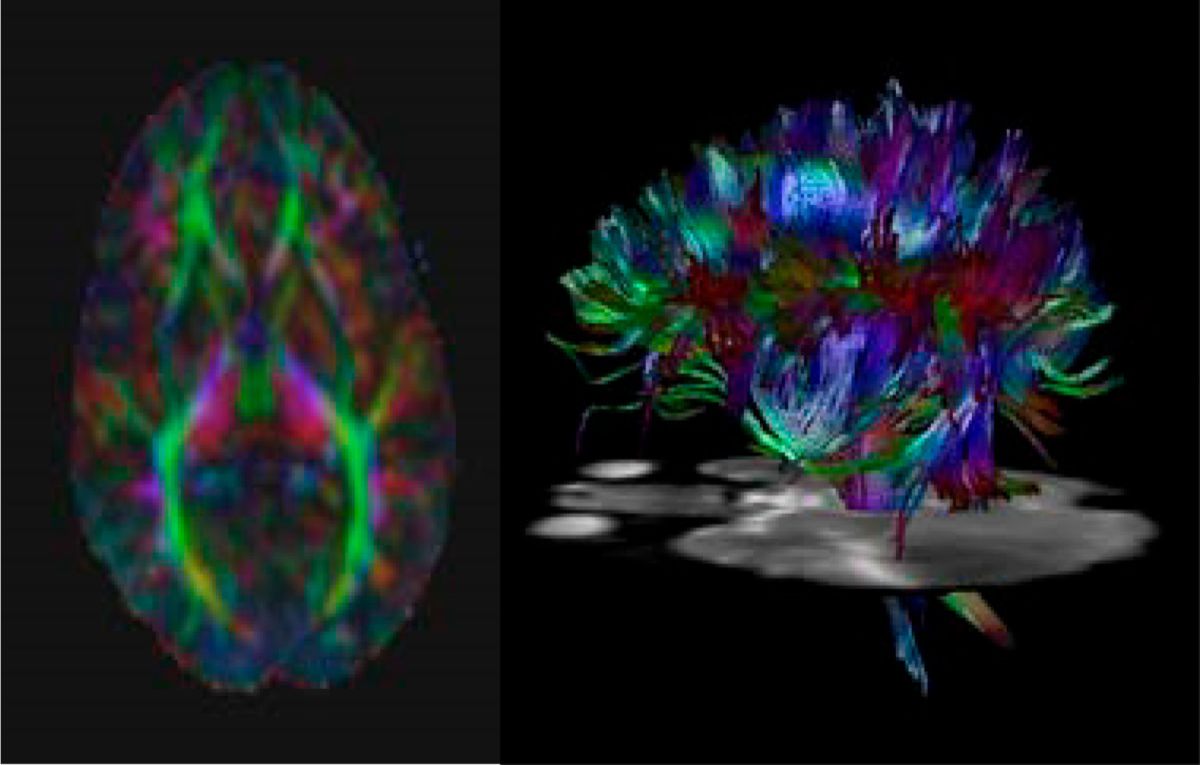
Diffusion Tensor Imaging Data: A color-coded anisotropy map (left) reveals white matter fiber orientation (red = right/left, green = ant/post, blue = sup/inf). The fiber pathways are reconstructed (right; color-coded as on the left).

DTI data are corrected for image distortions due to eddy currents [123] and static B0 errors. The diffusivity of the brain is analyzed using the conventional diffusion tensor model [124] and our generalization based on spherical deconvolution is implemented using the high angular resolution diffusion imaging (HARDI) method (2.5 mm isotropic resolution; 92 diffusion directions; b = 1000 s/mm^2^; less than 15 min) [125]. HARDI provides information on crossing and diverging white matter fibers that allow tracking algorithms to perform well, in contrast with tensor-based algorithms which often break down. Although whole-brain DTI data can be acquired in 60 s, data can be unreliable in voxels containing more than one fiber orientation. Therefore, we use an advanced method that has been found to improve brain connectivity measurements [125] to track fibers between cortical parcels, defined using FreeSurfer (Figure 4) [126], with the number of fibers connecting each pair of cortical parcels used to construct a connectivity matrix for each adolescent [127,128].

Probabilistic fiber tractography is performed using the FMRIB FDT toolbox, which uses Bayesian techniques to estimate the most probable location of a pathway between two seed points [129–132]. Fiber tracking is initiated from all voxels within each seed mask, and a multi-seed-mask approach in which anterograde and retrograde tracts are summed together; these methods are used to robustly characterize each tract within the fronto-limbic and fronto-striatal circuits, as relevant to the study aims. Tests for between-group differences are conducted using general linear models with p values estimated using permutation testing (FMRIB Randomise tool; 5000 permutations). Appropriate covariates are included in the model and data are corrected for multiple comparisons using a cluster-forming threshold [90,133].

We use tract-based spatial statistics (TBSS) to examine voxel-level differences in FA between groups [123,134] which compares FA values of each group on skeletons/centers of white matter fiber bundles in order to avoid errors due to mis-registration between subjects. This method has the advantage of determining if a specific white matter tract is altered entirely and identifying local changes along the tract, which has clinical/functional significance [90,130]. The EVA single subject FA map is used as the model template in order to enhance alignment with the digital white matter atlas from Johns Hopkins University (JHU ICBM-DTI-81) [135]. Post-hoc analyses of radial diffusivity and parallel diffusivity are performed to facilitate the interpretation of any between-group FA differences.

### Neuroimaging Tasks and Functional Analyses

Adolescents complete an Emotional Go/No-Go fMRI Task (EmoGnG), which targets emotional and cognitive processes, and allows us to measure responses in the fronto-limbic circuit [136]. Participants are presented with images of faces (calm, fearful or happy expressions) for 500 ms and asked to quickly and accurately respond to the “Go” stimuli and not to respond to the “No-Go” stimuli. Participant reaction times are measured with respect to targets (Go), defined by distinct emotional expressions, as well as their ability to withhold responses to non-targets (No-Go) (e.g., click only for fearful faces). Our version of the task includes six runs totaling 48 faces, with targets occurring in 75% of the trials, and with each 5-minute run comprising of the following conditions presented in a pseudorandom order: Happy-Neutral (HN), Neutral-Happy (NH), Sad-Neutral (SN), Neutral-Sad (NS), Fearful-Neutral (FN), and Neutral-Fearful (NF). Contrasts generated for analyses include responses (Go and NoGo), stimuli type (emotional expressions), and trial phase (early, middle, and late). EmoGnG has been used in prior studies to demonstrate functional connectivity between the AMG and PFC in response to negative emotional stimuli, such that increased activity in the PFC is associated with decreased AMG activity [136].

Participants also complete a Monetary Reward Task, which has been shown to reliably elicit striatal and medial PFC responses to anticipation and receipt of reward in both adolescent and adult subjects, including individuals with mood disorders [137–140]. Adolescents are instructed that they can win or lose money by guessing whether an upcoming card’s value is going to be high or low. Unknown to the participants, the outcome of each trial is predetermined and, of the 24 trials, there are 6 win, 6 loss, 6 no-win and 6 no-loss trials, all presented in a pseudorandom order. Contrasts generated for analyses include reward anticipation > baseline and reward win > baseline.

Rest and task-based images are aligned using rigid-body co-registration to reduce the effects of head motion and subsequently co-registered to the high-resolution T1-weighted structural image. The transformation of the structural image to atlas space is then applied to the functional images, followed by resampling, yielding functional images at 3mm isotropic resolution in the atlas space. These are spatially smoothed using a 6mm-FWHM Gaussian kernel. After initial pre-processing, time series at each voxel have white matter, cerebrospinal fluid and motion-related signals removed via regression [141], then are low-pass filtered at 0.1 Hz to retain low frequencies relevant for connectivity estimation. Head motion, which can be a significant confound in fMRI connectivity studies using adolescent participants [142], may be used as a potential covariate if it is found to differ between groups. To minimize potential confounds, we identify volumes that show large displacement (>0.5 mm) or large change in global signal (>0.5%) relative to the preceding volume, and remove them prior to conducting connectivity analyses [142].

**Figure 5.**
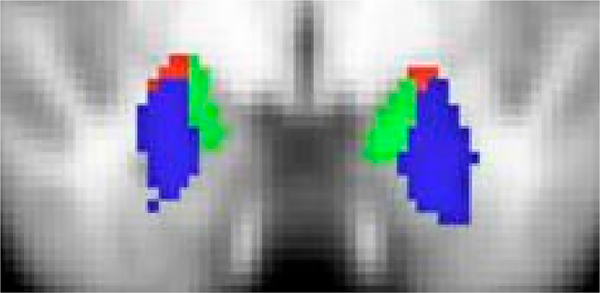
Amygdala Sub-regions (blue: centromedial; red: laterobasal nucleus; and green: superficial).

**Figure 6.**
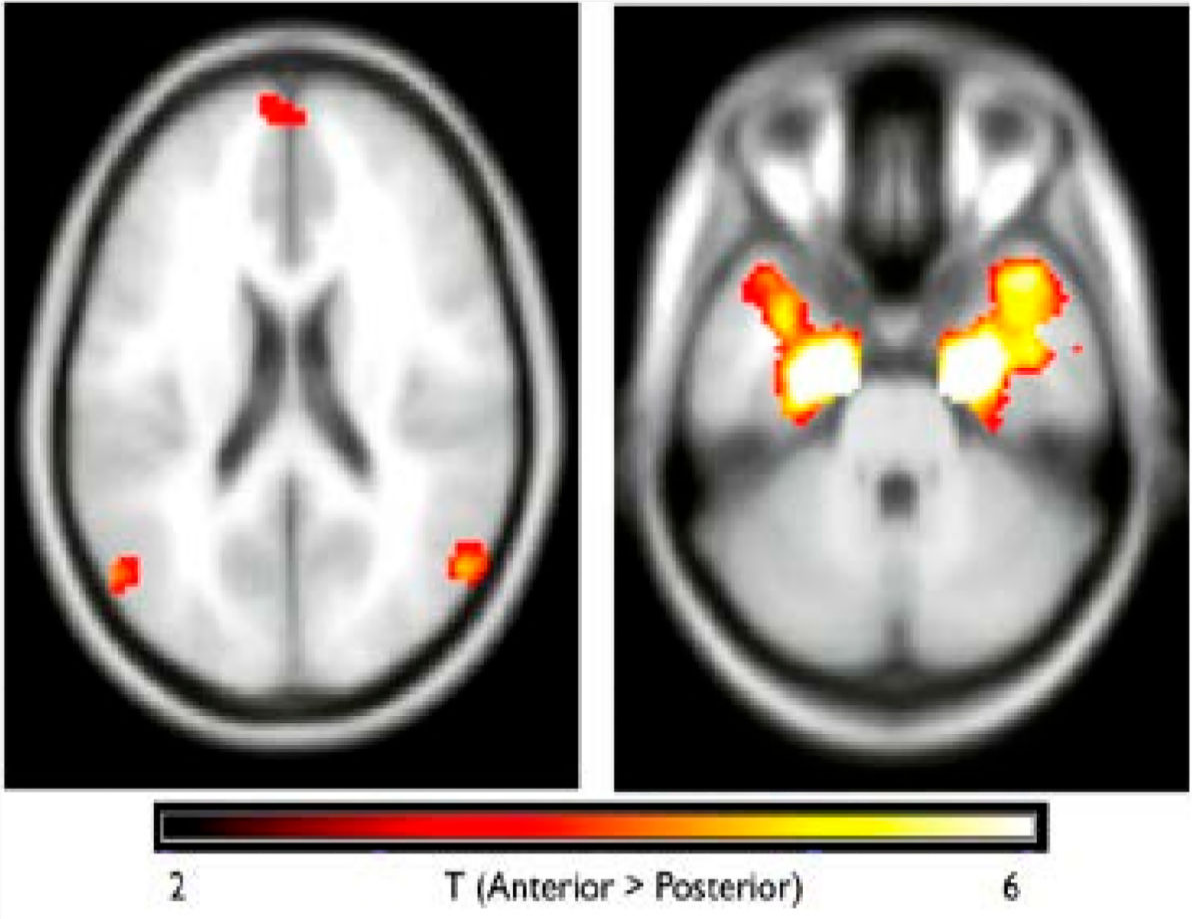
Functional Connectivity (FC): Anterior and posterior Hippocampus (HIPP) show different patterns of FC; anterior HIPP exhibits stronger FC with default network (left) and anterior temporal lobe (right).

Resting-state functional connectivity is calculated as Z-transformed correlation coefficient between pre-processed time series, using the CONN toolbox [143] and connectivity between seed region time series and each grey matter voxel is calculated to create connectivity maps for each seed region. In addition to the commonly used seed-based analysis, we utilize generalized psychophysiological interaction (gPPI) for task-based analyses, which allows us to understand how brain regions interact in a task-dependent manner with greater sensitivity and specificity than the standard PPI (sPPI) [144,145]. For both types of functional connectivity, we utilize seed-region approaches, defined using probabilistic atlases derived from structural tracings, with the following seeds: laterobasal, centromedial and superficial sub-regions of the AMG (Figure 5) [146–149], anterior and posterior regions of the HIPP (Figure 6) [150], and inferior and superior parts of the VS [151,152]. The resulting T maps are then thresholded at *p* < 0.05, using random field theory to correct for the multiple voxel comparisons based on spatial smoothness [153,154] to identify brain regions where the groups have differences in connectivity.

### Integration of Structural and Functional Analyses

Adult depression studies have shown the significant complimentary benefits of combining structural and functional neuroimaging techniques with respect to deepening our understanding of pathophysiology and treatment responsiveness [155,156]. Therefore, our multi-modal imaging methods enhance our ability to determine whether observed functional group differences indicate underlying structural changes, or if they are in fact distinct, thereby potentially uncovering the underlying mechanisms of brain changes in depressed adolescents. Correlations between grey matter density and functional activation are tested using the biological parametric mapping toolbox that provides voxel-level correlations between two imaging modalities [157]. We will use analysis of variance or covariance (ANOVA/ANCOVA) to test for group differences in the structure-function correlations across the whole brain, corrected for multiple comparisons (FWE < 0.05), a method previously used to demonstrate associations between grey matter volume and AMG responses to emotional faces [158].

Functional connectivity is tested by integrating structural and functional data using two different approaches. First, we compare groups on the correlation between overall strength of the structural connectivity (i.e., FA values) and functional connectivity (i.e., beta values) for the fronto-limbic tract (e.g., cingulum bundle and uncinate fasciculus) and the fronto-striatal tract (e.g., fronto-caudal). Values for each group are then r-to-z transformed and tested using ANOVA/ANCOVAs, thereby implementing a conservative test of structure-function association for the entire tract. Second, because there may be more specific relationships between structure and function, we use regression analyses to test for differences at each voxel within the path. Functional connectivity values, group, and group × functional connectivity interaction are all used as predictor variables. *P*-values are then estimated using permutation testing (FMRIB Randomise tool) and corrected for multiple comparisons using a cluster-forming threshold in FSL.

### Data Management and Statistical Analyses

Data management is completed through the HIPAA-compliant remote electronic data capture (REDCap) system [159], whereby respondents are able to complete self-report measures directly into the system under their respective participant ID numbers. Handling method of missing data will depend upon the sample size used in the analyses and the randomness status of missing variables [160]. Missing data are accounted for by including “missingness” as a covariate in analyses, and multiple imputation methods, for example, are used in variables when more than 5% are missing completely at random [161,162]. Primary variables used to test each study hypotheses are indicated in Table 1 as associated with each aim.

To test for the moderation effects of maltreatment on structural and functional alterations associated with MDD within the fronto-limbic circuit (Hypothesis 1) and fronto-striatal circuit (Hypothesis 2), multiple regressions will be used and will include MDD and MALTX as main effects and MDD × MALTX as an interaction term; demographic and clinical covariates will be included if these differ by group. Focus will be placed on simple main effects when interactions are significant. Subsequently, we will assess for structural and functional differences in the depressed samples between participants in the MDD and MDD + MALTX groups. Secondary analyses will include testing for differences between NC and MALTX (non-depressed adolescents), between MALTX and MDD + MALTX (maltreated adolescents), and between NC and MDD (non-maltreated adolescents).

In the event that analyses do not yield significant interactions, we will focus on the main effects of MDD and MALTX when testing group differences. Accordingly, we aim to answer the following questions with our findings: (a) whether the neural substrates of MDD participants differ from those without MDD, irrespective of abuse history; and (b) whether the neural substrates for MALTX participants differ from those without MALTX, irrespective of depression. Within-group analyses will include the covariates of clinical (e.g., depression severity, maltreatment severity) and demographic (e.g., age, sex/gender, SES) variables. Similar analyses will be conducted to test for the moderation effects of maltreatment on the association between distinct depressive symptom profiles and structural/functional connectivity measures in fronto-limbic and fronto-striatal circuits (Hypothesis 3). In these analyses, various domains of depression will either be dichotomized (e.g., anhedonia: yes/no) or tested as dimensional variables by mean centering the scores.

For exploratory analyses, we will examine the relationship between structural connectivity changes and functional connectivity, and between resting-state and task-based functional connectivity. In finding alterations in the key nodes common to cognitive-emotional and reward circuits, we seek to establish whether they influence both emotion- and reward-processing. Whole-brain analyses will be performed to identify changes in the extended parts of these circuits (e.g., bed nucleus of the stria terminalis, globus pallidus, thalamus). Various covariates will be included in our models as moderators on brain changes, such as age of depression onset, PTSD symptoms, and social support, and these will be examined in relation to behavioral and neurocognitive variables.

Based on power analyses utilizing traditional criteria (G power 3.1.9.2; alpha = 0.05 two-tailed, power = 80%) [163], our projected sample size of 240 adolescents within the four groups will allow us to detect an effect size of *f*^2^ = 0.05, indicating small-to-medium- effect sizes [164]. Given the recommended sample size of 40 per group in order to yield reliable estimates of group differences in fMRI studies [165], as well as the multiple variables of interest included in our protocol, we aim to enroll 60 adolescents per group.

### NIH Grant Reviewer Comments

The grant application received an Impact Score of 20 and a 6th Percentile Score. The main weaknesses expressed by the review committee include the unreliability of participants’ recollections about maltreatment history and the cross-sectional study design. Specifically, the cross-sectional design may not be able to reveal the dynamic effects of maltreatment and brain changes. Some reviewers indicated that the approach, in and of itself is not necessarily innovative, but the question is critical and significance of the study substantial. The committee indicated that these weaknesses did not diminish the reviewers’ enthusiasm and they agreed that the neural markers associated with the two depression subtypes may lead to better-informed treatments.

We acknowledge that retrospective reports of maltreatment are not very reliable. However, we instituted a multi-informant, multi-method assessment to improve reliability. Both youth and parent are interviewed using a standardized instrument (CAI). Additionally, we obtain information from Child Protective Services, when feasible. Youth also complete a self-report detailing past traumas (CTQ). Furthermore, given the shorter life history, retrospective bias is less in adolescents compared to adult samples.

We agree that prospective studies are the gold standard for identifying temporal changes in neurocircuitry in relation to maltreatment history, depression outcome and the associated neural changes, and we gave it considerable thought. However, given our current knowledge in this field and relative costs, we decided that the best course of action is a well-designed, cross-sectional study as the first step to distinguish unique and interactive effects of maltreatment and depression before embarking on a more expensive and intensive longitudinal study.

This study’s innovation is primarily conceptual. We felt it was important to use established methods to compare findings from existing data in adolescent/adult samples to distinguish the two depression phenotypes. We also use the latest advances in MRI data collection and analysis methods to provide exceptional resolution to facilitate distinction between the groups.

### Current Status

We are currently at the end of Year 2 of a 5-year project. As of March 2020, we recruited and completed 131 participants, with 24 adolescents in MDD + MALTX, 37 in MDD, 13 in MALTX, and 57 in NC. Only seven participants (not counting the 131 completed) were withdrawn thus far following informed consent due to various reasons (e.g., the age of onset of first incident of abuse; not meeting severity threshold for single CAI abuse item despite high total scores; MRI incompatibility; and scheduling conflicts). Additionally, of the adolescents screened, the most common reasons for ineligibility are MRI incompatibility (e.g., dental braces), current psychotropic medication, MDD history but no current episode, and personal or family history of bipolar disorder.

As per NIH mandate for this Award, we cannot ask for abuse history during the phone screen. We continue to make strides toward recruiting these high-risk youths, including reaching out to over 150 community organizations and agencies across Southern California, including agencies working with victims of trauma. The Governor of California has allocated $45 Million in the 2020–2021 fiscal year budget to reimburse Medicaid providers for screening of Adverse Childhood Experiences (ACEs) and an additional $50 Million to train primary care providers on administering these screenings (https://chronicleofsocialchange.org/child-welfare-2/californias-surgeon-general-readies-statewide-screening-for-child-trauma/37658). The California Department of Health Care Services has just implemented training to all Medi-Cal (Medicaid) providers on ACEs screening (https://www.dhcs.ca.gov/provgovpart/Pages/TraumaCare.aspx). With these new initiatives, we anticipate that we will be in a better position to recruit the MALTX and MDD + MALTX groups.

### Scientific Presentations

Preliminary results from the available data suggest structural and functional differences between MDD and MDD + MALTX as well as evidence of neurobiological changes in the MALTX group which do not manifest any psychiatric disorders [165–171].

- Thirion B, Pinel P, Meriaux S, Roche A, Dehaene S, Poline JB. Analysis of a large fMRI cohort: Statistical and methodological issues for group analyses. NeuroImage. 2007;35(1):105–20 [165].
- Van Erp T, Jirsaraie R, Faulkner M, Scambray K, Fong J, Taylor D, Rao U. Dentate gyrus volume is associated with childhood maltreatment and depression severity in adolescents. Presented at the 57th Annual Meeting of the American College of Neuropsychopharmacology; 2018 Dec 9–13; Hollywood, FL, USA [166].
- Faulkner M, Jirsaraie R, Zurita T, Fong J, Scambray K, Rao U. Unpredictability in childhood predicts executive function impairment in depressed and non-depressed adolescents. Presented at the 6th Annual Symposium Organized by the Conte Center at UCI; 2019 Mar 12; Irvine, CA, USA [167].
- Sharma A, Scambray K, Jirsaraie R, Faulkner M, Rao U. White matter changes in fronto-limbic pathways in adolescent depression. Presented at the 6th Annual Symposium Organized by the Conte Center at UCI; 2019 Mar 12; Irvine, CA, USA [168].
- Sharma A, van Erp TGM, Scambray K, Jirsaraie R, Faulkner M, Rao U. Sustained amygdala response to fearful faces in depressed adolescents with childhood maltreatment. Presented at the 66th Annual Meeting of the American Academy of Child and Adolescent Psychiatry, 2019 Oct 14–19; Chicago, IL, USA [169].
- Sharma A, van Erp TGM, Faulkner M, Forbes E, Rao U. Decreased striatal response to monetary reward in depressed adolescents. Presented at the 58th Annual Meeting of the American College of Neuropsychopharmacology; 2019 Dec 8–11; Orlando, FL, USA [170].
- Millwood SN, Gillespie M, Sharma A, Huszti H, Rao U. Amygdala volume differences in depressed adolescents with and without childhood maltreatment. Presented at the 7th Annual Symposium Organized by the Conte Center at UCI; 2020 Feb 25; Irvine, CA, USA [171].

## DISCUSSION

The current study aims to understand the unique contribution that maltreatment history can have on the neurobiology of adolescent depression, with the goal of identifying two distinct depression subtypes. We address current gaps in the neuroimaging literature by focusing on adolescents, reducing participant heterogeneity by using stringent depression and maltreatment criteria, using comprehensive psychodiagnostic interviews, and implementing state-of-the-art, multi-modal neuroimaging techniques. We expect to differentiate structural and functional alterations in fronto-limbic and fronto-striatal circuits between MDD and MDD + MALTX groups, and determine whether maltreatment history moderates the association between depressive symptom profiles and these neural circuits. Moreover, we expect to discover whether maltreatment-induced neural differences are distinct or whether they reflect associated alterations in brain structures. We will also assess whether individual differences in neural correlates among these groups serve as risk or resiliency markers for depression. Exploratory analyses will allow us to examine the correlations between various clinical and demographic variables and neural changes in the MDD + MALTX group. The findings from this study will broaden and deepen our understanding of the neurobiological correlates of adolescent MDD versus MDD + MALTX, which may have important implications for shaping clinical formulation and treatment guidelines, predicting treatment outcomes more effectively, and indexing treatment response for the two MDD subtypes. Such knowledge will also be helpful in developing new treatments for subgroups that do not respond well to traditional interventions. With well-characterized samples of adolescents and promising results from this cross-sectional study, we hope to follow these cohorts longitudinally with new intramural and/or extramural funding to better characterize the neurobiological vulnerability and resiliency factors associated with depression onset and recurrence during the developmental transition to adulthood.

## Figures and Tables

**Table 1.** Assessment schedule.

Assessment	Type	Informant		Visit	Purpose
		Youth	Caregiver		
Demographics	self-report	×	×	Screen	Eligibility
MALTX and psychiatric history	self-report	×	×	Screen	Eligibility
Additional demographic information	interview	×	×	1	Covariate; supplementary independent variable (Aims 1–3)
Tanner stage	self-report	×		1	Eligibility; covariate; supplementary independent variable (Aims 1–3)
Psychopathology (K-SADS-PL)	clinician	×	×	1	Eligibility; independent variable (Aims 1–2)
Depressive symptoms/severity (CDRS-R)	clinician	×		1	Covariate (Aims 1–2); supplementary dependent variable (Aim 3)
Depressive symptoms/severity (BDI)	self-report	×		1	Covariate (Aims 1–2); supplementary dependent variable (Aim 3)
Depressive symptoms/anhedonia (TEPS)	self-report	×		1	Dependent variable (Aim 3)
Depressive symptoms/rumination (CRSQ)	self-report	×		1	Dependent variable (Aim 3)
Mood/positive-negative valence (PANAS)	self-report	×		1	Dependent variable (Aim 3)
Anxiety symptoms (SCARED)	self-report	×	×	1	Covariate (Aims 1–3); independent variable (exploratory analysis)
PTSD symptoms (PCL-5)	self-report	×		1	Covariate (Aims 1–3); independent variable (exploratory analysis)
Family psychiatric history (FH-RDC)	clinician		×	1	Eligibility; covariate (exploratory analysis)
Childhood adversity (CAI)	interview	×	×	1	Eligibility; covariate (Aims 1–3); independent variable (exploratory analysis)
Child/adolescent trauma (CTQ)	self-report	×		1	Covariate (Aims 1–3; exploratory analysis)
Adolescent stress (ASQ)	self-report	×		1	Covariate (Aims 1–3); independent variable (exploratory analysis)
Social support (SSI)	self-report	×		1	Covariate (Aims 1–3); independent variable (exploratory analysis)
Social functioning (SAS-SR)	self-report	×		1	Independent variable (exploratory analysis)
Parent-child relationship (PBI & C-PRS)	self-report	×	×	1	Covariate (Aims 1–3); independent variable (exploratory analysis)
Questionnaire of Unpredictability in Childhood	self-report	×	×	1	Covariate (Aims 1–3); independent variable (exploratory analysis)
Autistic traits (SCDC)	self-report		×	1	Eligibility
Handedness (Edinburgh Scale)	self-report	×		1	Covariate (Aims 1–3; exploratory analysis)
MRI safety screen	self-report	×	×	1	Eligibility for neuroimaging
Neurocognitive battery	assessor	×		2	Dependent variable (exploratory analysis)
Neuroimaging (mock scan)	task	×		2	Eligibility for neuroimaging
Neuroimaging (sMRI, DTI, rs-fMRI, fMRI)	task	×		3	Dependent variable (Aims 1–3; exploratory analysis)

Note: K-SADS-PL: Kiddie Schedule for Affective Disorders and Schizophrenia – Present and Lifetime Version; CAI: Childhood Adversity Interview; CDRS-R: Children’s Depression Rating Scale–Revised Version; BDI: Beck Depression Inventory; TEPS: Temporal Experience of Pleasure Scale; CRSQ: Children's Response Style Questionnaire; PANAS: Positive and Negative Affect Schedule; SCARED: Screen for Child Anxiety-Related Emotional Disorders; PCL-5: PTSD Checklist for DSM-5; FH-RDC: Family History-Research Diagnostic Criteria; CTQ: Childhood Trauma Questionnaire; ASQ: Adolescent Stress Questionnaire; SSI: UCLA Social Support Inventory; SAS-SR: Social Adjustment Scale-Self Report; PBI: Parental Bonding Instrument; C-PRS: Child-Parent Relationship Scale; SCDC: Social and Communication Disorders Checklist; sMRI: structural magnetic resonance imaging; DTI: diffusion tensor imaging; rs-fMRI: resting state functional MRI; fMRI: functional MRI.
